# Let-7i-3p inhibits the cell cycle, proliferation, invasion, and migration of colorectal cancer cells via downregulating CCND1

**DOI:** 10.1515/med-2022-0499

**Published:** 2022-06-07

**Authors:** Fei Tu, Mengfan Li, Yinyu Chen, Huiru Chu, Shujie Wang, Lun Hai, Ting Xie, Fangfang Geng, Tiesuo Zhao, Qingzhi Wang, Zhiwei Feng

**Affiliations:** Department of Anatomy, Histology & Embryology, School of Basic Medical Sciences, Xinxiang Medical University, Xinxiang, China; Institute of Precision Medicine, Xinxiang Medical University, Xinxiang, China; School of Forensic Medicine, Xinxiang Medical University, Xinxiang, China; The First Affiliated Hospital of Xinxiang Medical University, Weihui, China; Department of Immunology, School of Basic Medical Sciences, Xinxiang Medical University, Xinxiang, China

**Keywords:** let-7i-3p, CCND1, proliferation, invasion, migration, colorectal cancer cells

## Abstract

Dysregulated microRNAs are closely related to the malignant progression of colorectal cancer (CRC). Although abnormal let-7i-3p expression has been reported in various human cancers, its biological role and potential mechanism in CRC remain unclear. Therefore, the purpose of this study was to investigate the expression and regulation of let-7i-3p in CRC. Here, we demonstrated that let-7i-3p expression was significantly downregulated in three CRC cell lines while CyclinD1 (CCND1) was upregulated compared with the normal colon epithelial FHC cells. Moreover, bioinformatics and luciferase reporter assays revealed that CCND1 was a direct functional target of let-7i-3p. In addition, let-7i-3p overexpression or CCND1 silencing inhibited cell cycle, proliferation, invasion, and migration and diminished the activation of p-ERK in HCT116 cells. However, exogenously expressing CCND1 alleviated these effects. Taken together, our findings may provide new insight into the pathogenesis of CRC and let-7i-3p/CCND1 might function as new therapeutic targets for CRC.

## 1 Introduction

Colorectal cancer (CRC) poses a serious threat to human life and health as it is the third most common cancer in the world and the fourth leading cause of cancer death [1,2]. Patients with advanced colorectal cancer cannot receive surgical treatment due to the development of liver and lung metastases [3,4]. Therefore, it is necessary to elucidate the pathogenesis and potential molecular mechanisms of CRC tumor metastasis, which will help to find potential therapeutic targets for CRC.

MicroRNAs (miRNAs) are a class of endogenous regulatory non-coding RNAs found in eukaryotes with a length of about 20–25 nucleotides [5]. miRNAs can downregulate the expression of target genes by inhibiting mRNA cleavage or translation repression [6,7]. In recent years, a large number of studies have shown that miRNAs are involved in a variety of cell processes, such as cell proliferation, invasion, migration, and cell cycle progression. For example, miR-BART10-3p regulates EBVaGC cell proliferation and migration by directly targeting DKK1 [8]. Transient activation of miR-294 leads to myocyte cell cycle reactivation [9]. Let-7i downregulates GREB1 to inhibit the progression of esophageal cancer [10]. Let-7i inhibits gastric cancer invasion and metastasis by targeting COL1A1 [11]. Interestingly, the microarray data of previous studies have shown that let-7i-3p levels are significantly reduced in CRC cell lines (SW620, LoVo) compared to normal colon epithelial FHC cells [12]. However, the molecular mechanisms and specific biological functions of let-7i-3p in CRC remain largely unknown.

Cyclin D1 is encoded by the CCND1 gene and is a promoter of the cell cycle, which is involved in the tumorigenesis of many cancers [13,14,15]. Previous studies have shown that high levels of CCND1 are associated with poor prognosis in CRC patients [16,17,18]. Therefore, understanding the regulatory mechanisms of CCND1 may help to develop strategies to combat colorectal cancer cell migration and invasion.

Based on the above considerations, this study aimed to investigate whether let-7i-3p regulates the cell cycle, proliferation, migration, and invasion of colorectal cancer cells by targeting CCND1.

## 2 Materials and methods

### 2.1 Cell lines and cell culture

The human colorectal cancer cell lines SW480, HCT116, LoVo, RKO, and HT29 and the normal colon epithelial cell lines FHC and 293T were obtained from the Chinese Academy of Sciences (Shanghai, China). All cells were cultured in Dulbecco’s modified Eagle’s medium (DMEM) (Gibco, USA) containing 10% fetal bovine serum (FBS) and 1% antibiotics (100 U mL^−1^ penicillin and 100 mg mL^−1^ streptomycin). They were incubated in a humidified atmosphere at 37°C containing 5% CO_2._

### 2.2 Oligonucleotide transfection

Let-7i-3p mimic (named as let-7i-3p), negative control duplex (named as NC), and siRNA against CCND1 (named as siCCND1) were synthesized by GenePharma (Shanghai, China) and were applied for transfection. Oligonucleotide transfection was performed using Lipofectamine 3000 reagents (Invitrogen, Carlsbad, CA, USA) according to the manufacturer’s protocol. The sequences are listed in Table 1.

**Table 1:** Oligonucleotide sequences

Name^a^	Sequence (5′–3′)^b^	Usage
let-7i-3p (sense)	CUGCGCAAGCUACUGCCUUGCU	Transfection
NC (sense)	UUCUCCGAACGUGUCACGUTT	Transfection
siCCND1-1 (sense)	CGCUGGAGCCCGUGAAAAATT	Transfection
siCCND1-2 (sense)	CCAGAGUGAUCAAGUGUGATT	Transfection
U6-F	CTCGCTTCGGCAGCACA	qRT-PCR
U6-R	AACGCTTCACGAATTTGCGT	qRT-PCR
CCND1-F	ATCAAGTGTGACCCGGACTG	qRT-PCR
CCND1-R	CTTGGGGTCCATGTTCTGCT	qRT-PCR
let-7i-3p-RT	GTCGTATCCAGTGCAGGGTCCGAGGTATTCGCACTGGATACGACAGCAAG	RT
let-7i-3p-Q	CGCTGCGCAAGCTACTGC	qRT-PCR
GAPDH-F	AAATCCCATCACCATCTTCC	qRT-PCR
GAPDH-R	TCACACCCATGACGAACA	qRT-PCR
CCND1-wt-F	CCGGAGCTCTTCAACCCACAGCTACTTGG	Plasmid construction
CCND1-wt-R	CCCGTCGACTCAGATGACTCTGGGAAACG	Plasmid construction
CCND1-mut-F	AGGCTGGTGGGAACTCGCCGGGGCACAGCGGAGTCT	Mutagenesis
CCND1-mut-R	GCGAGTTCCCACCAGCCTTTGGCCTCTCGATAC	Mutagenesis
CCND1-FL-F	CGCGGATCCATGGAACACCAGCTCCTGTG	Plasmid construction
CCND1-FL-R	CCGCTCGAGTCAGATGTCCACGTCCCGC	Plasmid construction

^a^F, forward primer; R, reverse primer.

^b^Mutated target sites are underlined.

### 2.3 RNA extractions and qRT-PCR

Total RNA was extracted from cells using the TRIzol reagent (Invitrogen, CA, USA) following the manufacturer’s protocol. Then, we used FastKing RT Kit (with gDNase) (TIANGEN, China) to synthesize cDNA. qRT-PCR was performed on a Quant Studio5 Real-time PCR System (Applied Biosystems, USA) with ChamQ™ Universal SYBR^®^ qPCR Master Mix (Vazyme Biotech, Nanjing, China).

### 2.4 miRNA expression

miRNA expression was measured using miRNA Universal SYBR^®^ qPCR Master Mix Assays (Vazyme Biotech, Nanjing, China). The reverse transcription reaction was performed with the miRNA 1st-Strand cDNA Synthesis Kit (by stem-loop) (Vazyme Biotech, Nanjing, China) according to the manufacturer’s protocol. Quantitative real-time PCR was performed on a Quant Studio5 Real-time PCR System (Applied Biosystems, USA). Amplification data were normalized to endogenous U6 expression. All procedures were carried out in triplicate and the relative expression was calculated by the 2^−ΔΔCT^ method.

### 2.5 Plasmid construction and dual-luciferase assay

The fragment of the 3′-UTR of CCND1 containing the predicted let-7i-3p-binding site was amplified by PCR and inserted between the SacI and SaII restriction sites of the pmirGLO Dual-Luciferase miRNA Target Expression Vector (kindly provided by Prof. Qifa Li of Nanjing Agricultural University, Nanjing, China). For mutation, the let-7i-3p-binding motif in the 3′-UTR of the CCND1 gene was mutated by using the Mut Express MultiS Fast Mutagenesis Kit V2 (Vazyme Biotech, Nanjing, China). Luciferase activity was measured 24 h after transfection using the Dual-Glo luciferase assay system (Promega, USA). Renilla luciferase activity served as the internal control. The CCND1 cDNA was amplified by PCR and cloned into the pcDNA3.1 (+) (kindly provided by Prof. Qifa Li of Nanjing Agricultural University, Nanjing, China). All of the constructs were verified by sequencing.

### 2.6 Western blot analysis

The cell pellets were harvested and re-suspended in lysis buffer (20 mM Tris–HCl, pH 7.4, 150 mM NaCl, 1% Triton X-100, 25 mM β-glycerol-phosphate, 1 mM Na_3_VO_4_, 10% glycerol, 1× PMSF, with the sigma phosphatase inhibitors and protease inhibitor; Pierce, Rockford, IL, USA). The re-suspended cell pellet was then incubated on ice for 20 min, followed by centrifugation at 12,000×*g* for 20 min at 4°C. The supernatants were collected and protein concentrations were measured using the BCA Protein Assay Kit (Beyotime, Shanghai, China). Finally, cell lysates were subjected to western blot analysis of the following antibodies: CCND1 (CST, 55506S), α-tubulin (CST, 3873S), p-Erk1/2 (CST, 4370S), Erk1/2 (CST, 4695S), and GAPDH (protein-tech, 60004-1).

### 2.7 Cell proliferation assay

Cell Counting Kit-8 (CCK-8) (Beyotime, Shanghai, China) was used to measure the cell proliferation according to the manufacturer’s recommendations. Cells were transfected with let-7i-3p mimic or mimic NC, siCCND1, or pcDNA-CCND1 + let-7i-3p mimic. Forty-eight hours later, the transfected cells were trypsinized, counted, and replated at a density of 2000 cells/well in a 96-well plate, 10 μL of CCK-8 solution was added into the medium at different time points, and the absorbance (450 nm) was assessed on a SpectraMax iD3 Multi-Mode Microplate Reader (Molecular Devices, USA). All the experiments were performed at least three times, and the mean was calculated.

### 2.8 Colony formation assay

The transfected cells as described above were plated in a six-well plate (1,000 cells per well) and cultured with DMEM for about 2 weeks. Proliferating colonies resulting from the surviving cells were fixed with 3.7% methanol, stained with 0.1% crystal violet, and counted. Colonies containing at least 50 cells were scored. Each assay was performed in triplicate.

### 2.9 Cell cycle and apoptosis assays by flow cytometry

For cycle analysis, the test was performed using a cell cycle and apoptosis analysis kit (C1052; Beyotime, Shanghai, China). For apoptosis analysis, the test was performed using the Annexin-V-PE/7-AAD apoptosis detection kit (Vazyme Biotech, Nanjing, China). The transfected cells were harvested, washed, and stained according to the manufacturer’s protocol. Then, the stained cells were measured by CytoFLEX (Beckman Coulter, USA) and analyzed using FlowJo software version 7.6. Three independent assays were conducted.

### 2.10 Scratch wound-healing assay

A scratch wound-healing assay was performed for the analysis of cell migration. The transfected cells were incubated on six-well plates (3 × 10^5^ cells per well) with 5% CO_2_ at 37°C. After 24 h, the plate was scratched using a pipette. Then, the cells were washed and incubated with fresh serum-free DMEM in the incubator and observed at 0 and 48 h. Images were acquired under an inverted microscope. Experiments were performed in triplicate.

### 2.11 Cell invasion assay

For the invasion assay, the transfected cells were put into the upper chamber of each well of a 24-well transwell polycarbonate membrane (8 μm pore size, millepore) coated with Matrigel (BD, USA). Medium containing 10% FBS, which served as a chemoattractant, was put into the lower chambers. After wells were incubated for 24 h at 37°C, the surface of cells on the upper membrane was removed. The cells were fixed and stained with 0.05% crystal violet. Six random fields of each chamber were photographed using an inverted microscope at 200× magnification. The mean of triplicate assays for each experimental condition was used.

### 2.12 Statistical analysis

All measurement data are represented as mean ± standard deviation. The statistical differences between groups were analyzed using *t*-tests of GraphPad Prism9. *P*-values were determined by paired-samples *t*-tests: *P < 0.05, **P < 0.01, and ***P < 0.001.

## 3 Results

### 3.1 Let-7i-3p is significantly downregulated but CCND1 upregulated in CRC cells

**Figure 1:**
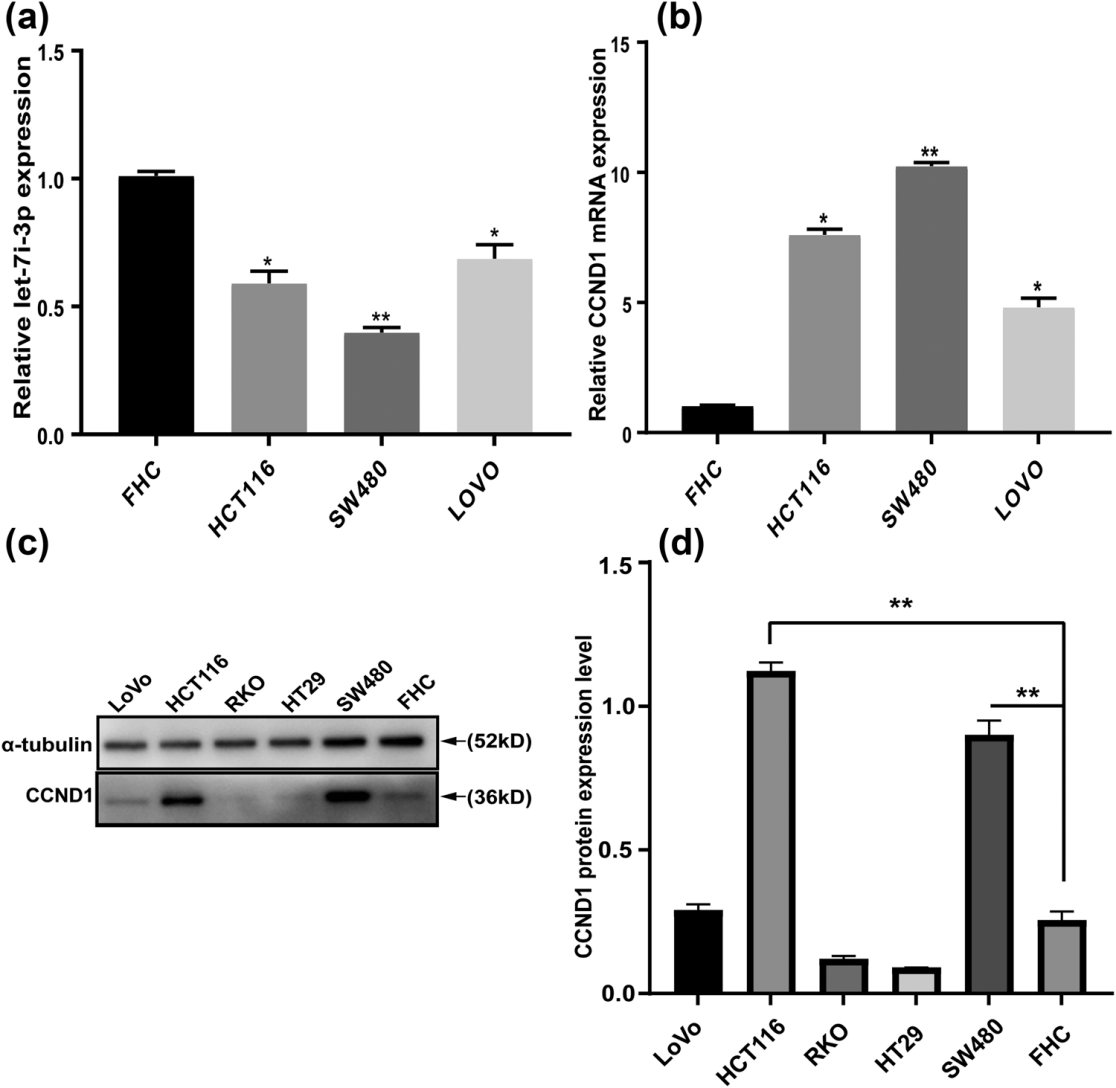
The expression of let-7i-3p and CCND1 in CRC cells. (a) The expression of let-7i-3p in four cell lines was determined by qRT-PCR. (b) The expression of CCND1 mRNA in four cell lines. (c) The expression of CCND1 protein in six cell lines was determined by western blot. (d) Quantification of CCND1 was normalized to α-tubulin. *P < 0.05 and **P < 0.01.

To verify whether let-7i-3p was abnormally expressed in CRC cells, we analyzed the expression level of let-7i-3p in three CRC cells (HCT116, SW480, and LoVo) and normal colon epithelial cell line (FHC) (Figure 1a). Consistent with the microarray data of previous reports [12], our qRT-PCR results showed that the expression of let-7i-3p in CRC cells was significantly reduced compared with FHC cells. Considering that let-7i-3p mainly plays a role through its target genes, we screened potential target genes by RNAhybrid. Comprehensive data analysis and literature review predicted that CCND1 might be a putative target gene of let-7i-3p. To evaluate the relation between let-7i-3p and CCND1 expression levels in CRC. Similarly, we detected CCND1 expression levels in three CRC cells and FHC. As shown in Figure 1b, the expression of CCND1 was upregulated in CRC cells compared with FHC cells. Next, CCND1 expression was detected in CRC cells and FHC by western blot. The results showed that the relative expression of CCND1 protein level in CRC cells (HCT116, SW480) was significantly upregulated compared with FHC cells (Figure 1c and d). These results further supported CCND1 as a potential target gene regulated by let-7i-3p.

### 3.2 Let-7i-3p inhibits cell cycle, proliferation, migration, and invasion in HCT116

**Figure 2:**
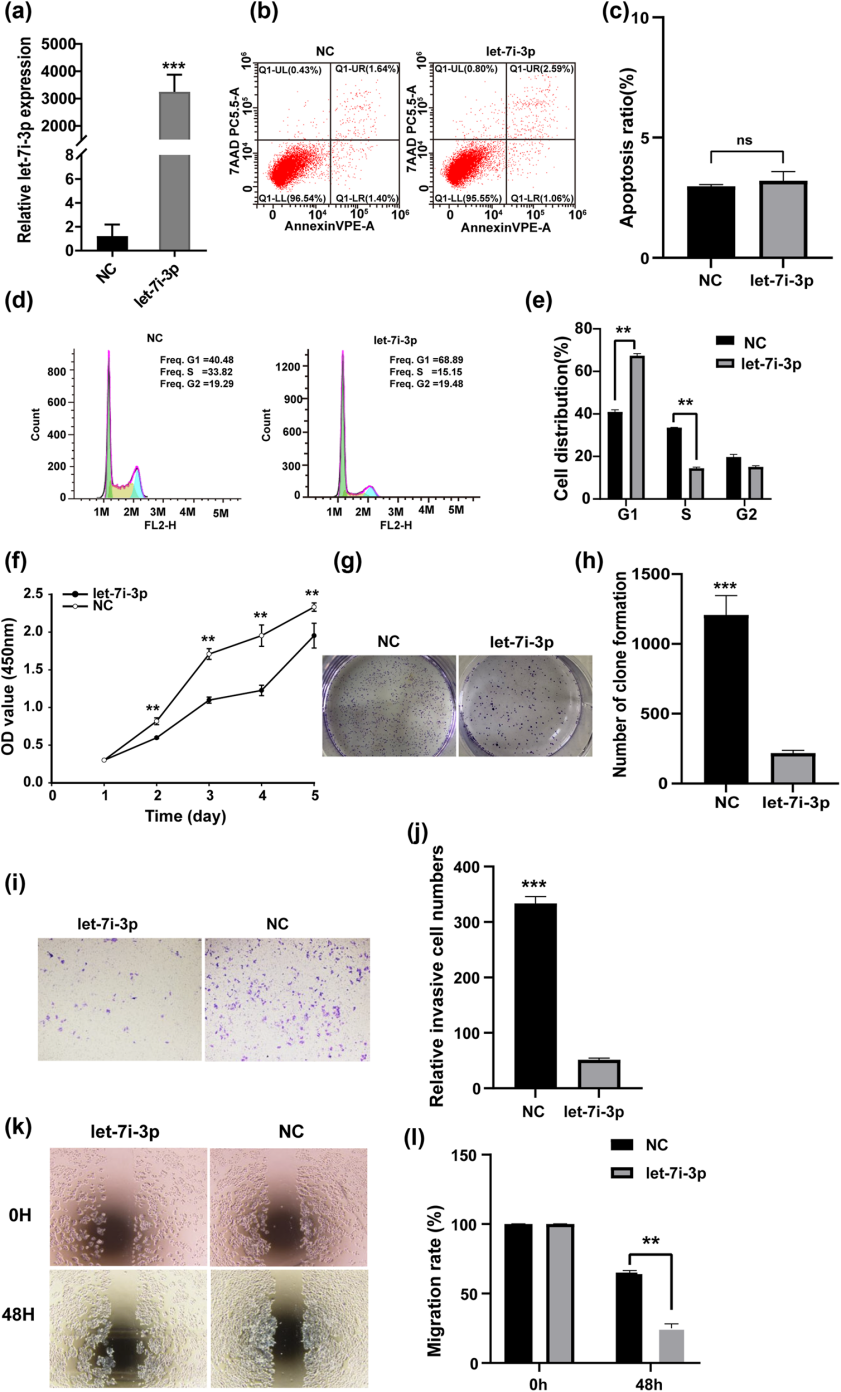
Let-7i-3p inhibits the cell cycle, proliferation, migration, and invasion but does not affect the apoptosis in HCT116. (a) The expressions of let-7i-3p were measured after transfecting let-7i-3p or NC into HCT116 cells. (b and c) Cell viability was determined by Annexin-V/7-AAD staining. Representative flow cytometric analysis of apoptosis (b) and statistical histogram was shown at right (c). (d) Relative cell cycle distribution detected by flow cytometry and statistical histogram was shown at right (e). (f) The effects of let-7i-3p mimics or NC on HCT116 cells’ proliferation as determined by CCK-8 assay. (g) A colony formation assay was used to detect the cell colony formation ability after the transfection of let-7i-3p in HCT116 cells and a statistical histogram was shown at right (h). (i) The effects of let-7i-3p mimics and NC on HCT116 cells’ invasion determined by transwell assay and statistical histogram was shown at right (j). (k) Images were acquired at 0 and 48 h after wounding. The percentage of the wound healing was calculated as (the width of wound at 0 h − the width of wound at 48 h)/the width of wound at 0 h and a statistical histogram was shown at right (l). *P < 0.05, **P < 0.01, and ***P < 0.001.

To examine the potential roles of let-7i-3p in CRC cells, the HCT116 was transfected with let-7i-3p or NC. Following transfection, the expression of let-7i-3p was significantly increased in the HCT116 transfected with the let-7i-3p (Figure 2a; *P* < 0.001). To verify the biological function of let-7i-3p in HCT116 cells, CCK8 assay, colony formation assay, flow cytometry, wound-healing migration assay, and transwell assay are performed. No difference was detected from Annexin-V/7-AAD staining, suggesting that let-7i-3p had no impact on apoptosis (Figure 2b and c). However, compared to the control group, significantly more cells were detected in the G1 phase, while fewer cells were detected in the S phase in the let-7i-3p-overexpressed cells (Figure 2d and e). Moreover, cck-8 and colony assay demonstrated that the cell proliferation capacity of HCT116 cells was remarkably suppressed (Figure 2f and h). In addition, as shown in Figure 2i–l, the width of the wound was significantly broader in let-7i-3p-overexpressed CRC cells compared with that in the control cells. In line with the wound-healing assay data, the results of the transwell assay indicated that overexpression of let-7i-3p inhibited the invasiveness of HCT116 cell. Moreover, let-7i-3p played the same role in the SW480 (Figure S1). Taken together, these data indicated that overexpression of let-7i-3p inhibited cell cycle, proliferation, migration, and invasion, but not apoptosis in CRC cells.

### 3.3 CCND1 is a direct target of let-7i-3p

**Figure 3:**
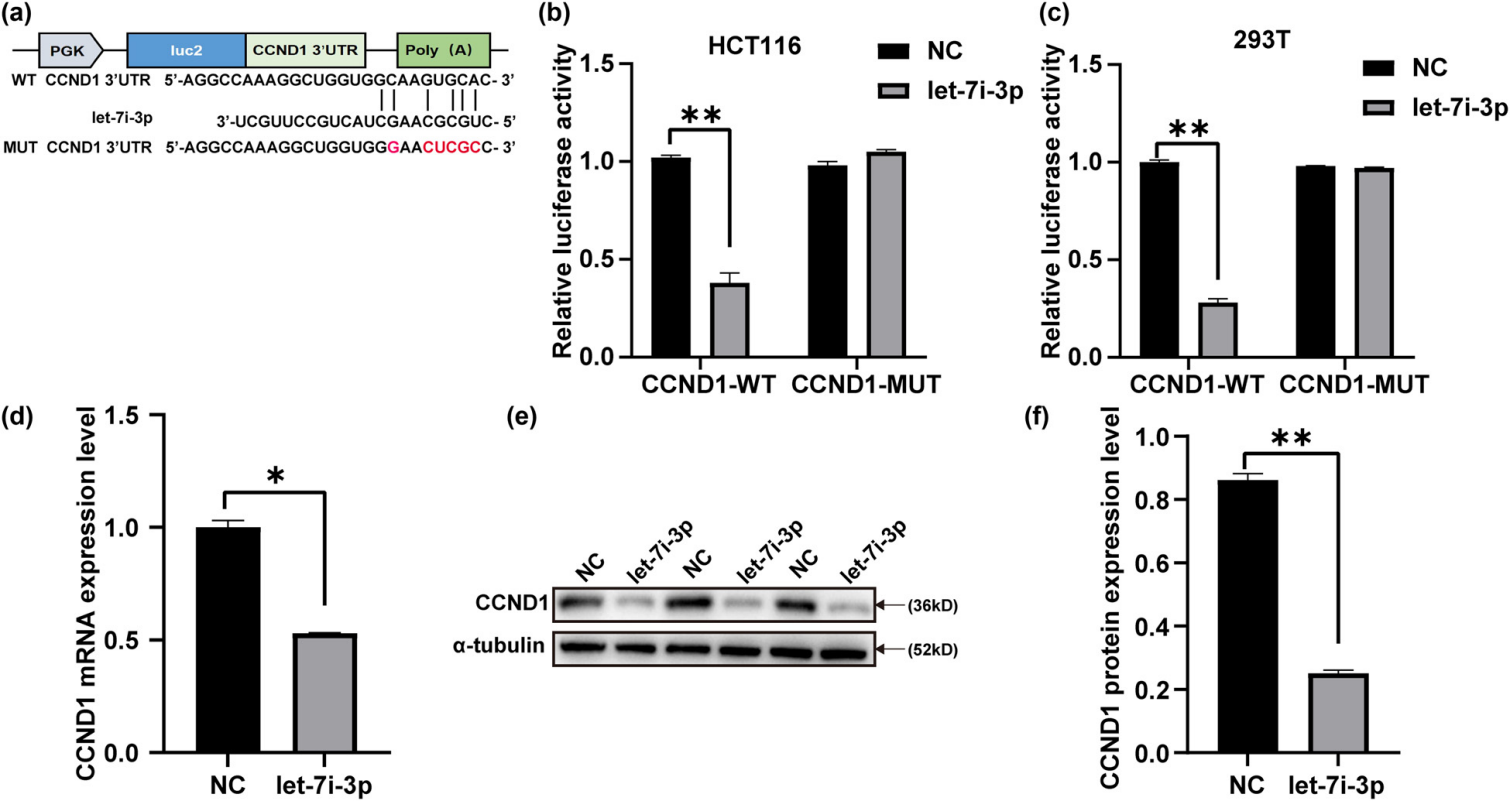
Let-7i-3p directly targets CCND1. (a) Putative WT and mutant‐type (MUT) let-7i-3p target sequences of CCND1 mRNA 3′-UTR. (b and c) Relative luciferase activity in HCT116 and 293T cells co-transfected with constructs carrying WT or mutant-type CCND1 mRNA 3′-UTR with let-7i-3p or NC. (d and e) The overexpression of let-7i-3p in the HCT116 cells attenuated the expression of CCND1 mRNA and protein levels, respectively, and a statistical histogram was shown at right (f). *P < 0.05 and **P < 0.01.

RNA-hybrid was used to analyze the potential let-7i-3p target gene. We constructed a dual-luciferase reporter plasmid recombined with either wild-type (WT) or mutant (MUT) type 3′-UTR of CCND1 (Figure 3a). The result showed that co-transfection of let-7i-3p significantly decreased the luciferase activity in HCT116 and 293T cells with WT 3′-UTR of CCND1 but not in those with MUT type (Figure 3b and c). To verify that CCND1 is the true downstream target of let-7i-3p, we examined the effect of the let-7i-3p expression on CCND1 expression by qRT-PCR and western blot. As shown in Figure 3d–f, let-7i-3p significantly suppressed both mRNA and protein expression levels of CCND1. These data suggested that CCND1 was a direct target of let-7i-3p.

### 3.4 Knockdown of CCND1 inhibits cell cycle, proliferation, migration, and invasion in HCT116

**Figure 4:**
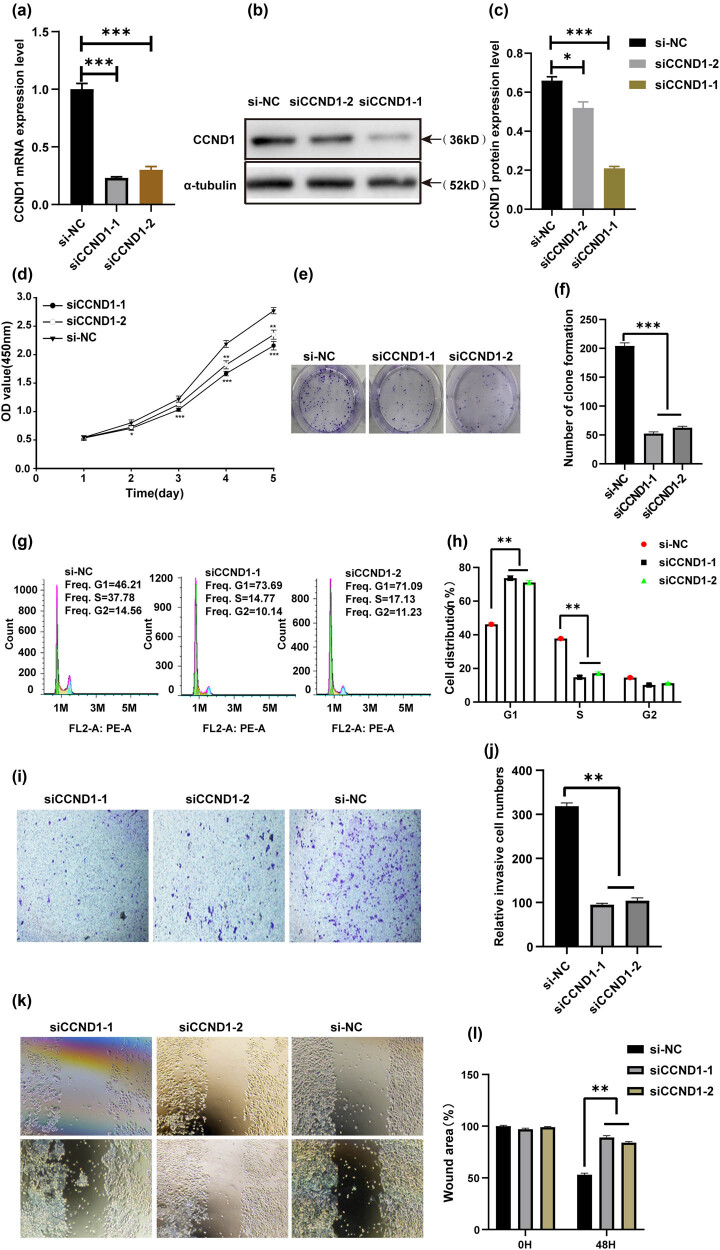
siCCND1 inhibits the proliferation, cell cycle, migration, and invasion of HCT116 cells. (a) QRT-PCR was used to detect the mRNA expression of CCND1 in siCCND1 and siNC. (b) Western blot was used to detect the protein expression of CCND1 in si-CCND1 and siNC and a statistical histogram was shown at right (c). (d) CCK-8 assay was used to detect the HCT116 cell viability after the knockdown of CCND1. (e) A colony formation assay was used to detect the cell colony formation ability after the knockdown of CCND1 and a statistical histogram was shown at right (f). (g) Cell cycle distribution was measured by flow cytometry and a statistical histogram was shown at right (h). (i) Transwell assay was used to detect the invasion of HCT116 cells after knocking down CCND1 and a statistical histogram was shown at right (j). (k) The change in cell migration was examined by wound-healing assay in HCT116 cells after knocking down CCND1 and a statistical histogram was shown at right (l). *P < 0.05, **P < 0.01, and ***P < 0.001.

To investigate whether siCCND1 has a similar function to let-7i-3p in CRC cells, two siCCND1s were transfected into HCT116 cells. The silencing of CCND1s was confirmed by real-time RT-PCR and western blot. The result showed that the siCCND1s’ transfection of HCT116 cells efficiently knocked down CCND1 mRNA and protein expression (Figure 4a and c). To further explore the role of CCND1 in HCT116 cells, we analyzed the effect of siCCND1 in controlling cell proliferation, cell cycle, migration, and invasion. The results showed that the silencing of CCND1 led to the significant inhibition of proliferation, migration, and invasion, in a pattern similar to that of let-7i-3p overexpression (Figure 4d and l).

### 3.5 CCND1 overexpression reverses the effects of let-7i-3p on HCT116 cells

**Figure 5:**
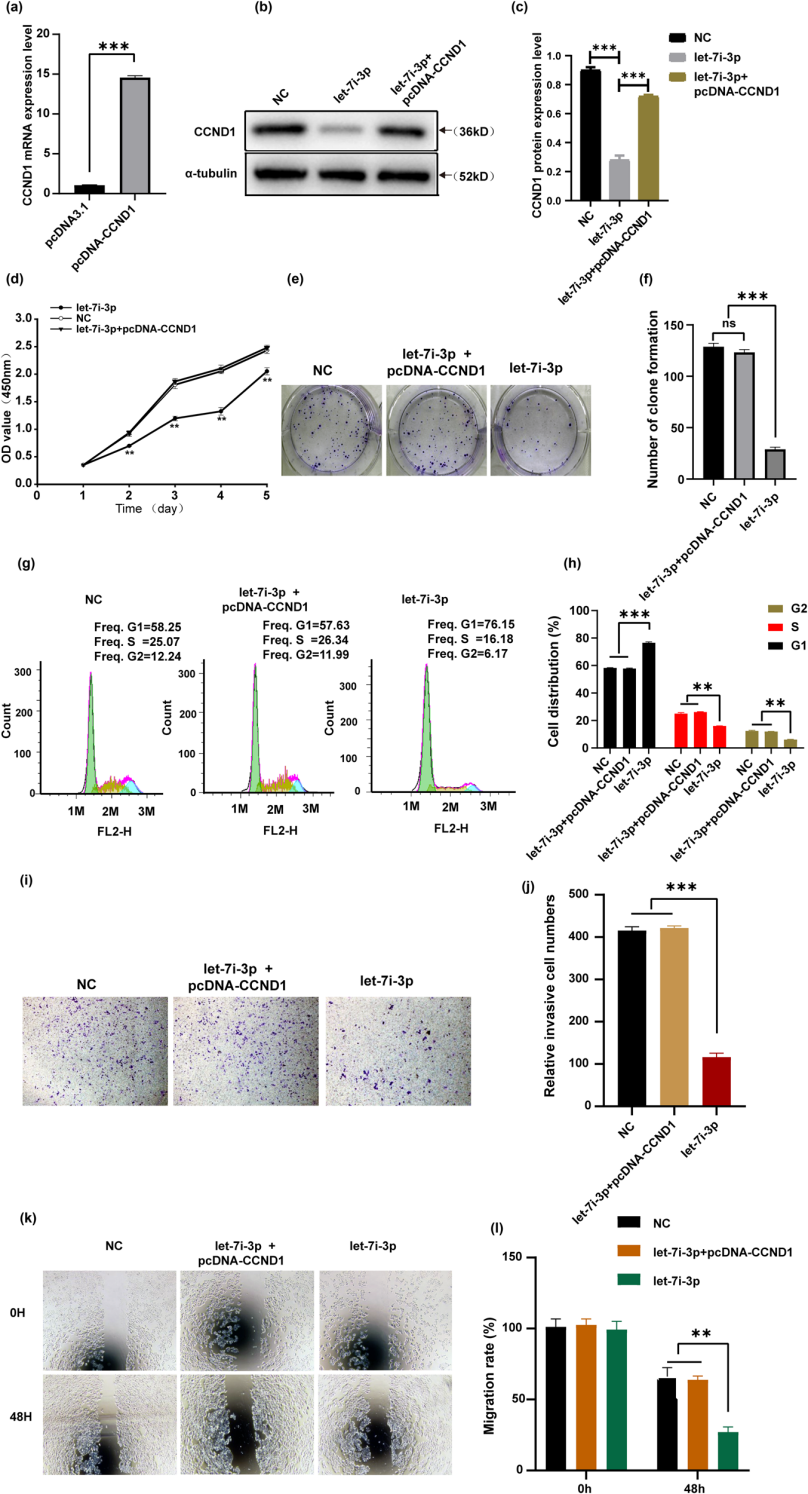
Overexpressed CCND1 could reverse the effects of let-7i-3p on HCT116 cells. (a) The level of CCND1 mRNA expression was detected by RT-PCR. (b) The level of CCND1 protein expression was detected by western blot and a statistical histogram was shown at right (c). (d) CCK-8 assay was used to explore the proliferation of HCT116 cells. (e) A colony formation assay was conducted to verify that ectopic CCND1 expression could reverse proliferation induced by let-7i-3p overexpression in HCT116 cells and a statistical histogram was shown at right (f). (g) Cell cycle distribution was measured by flow cytometry and a statistical histogram was shown at right (h). (i) Transwell assay was carried out to confirm the effects of CCND1 alteration in the invasion of HCT116 cells and a statistical histogram was shown at right (j). (k) The change in cell migration was examined by wound-healing assay in HCT116 cells and a statistical histogram was shown at right (l). *P < 0.05, **P < 0.01, and ***P < 0.001.

To confirm the function of CCND1 in CRC cells, we tested the effect of CCND1 overexpression on the cell cycle, proliferation, migration, and invasion. We ectopically expressed CCND1 together with let-7i-3p in HCT116 cells. qRT-PCR and western blot analyses showed that CCND1 mRNA and protein levels dramatically increased in pcDNA-CCND1-transfected HCT116 cells (Figure 5a and c). Furthermore, we performed CCK8 assay, colony-forming assay, flow cytometry, transwell assay, and wound-healing migration assay in HCT116 cells. As expected, the results showed that there was no significant difference between the pcDNA-CCND1 + let-7i-3p group and the control group (Figure 5d and l). Taken together, these results displayed that let-7i-3p inhibited cell cycle, proliferation, migration, and invasion in HCT116 by targeting CCND1.

### 3.6 Let-7i-3p decreases the ERK signaling pathway by downregulation of CCND1

**Figure 6:**
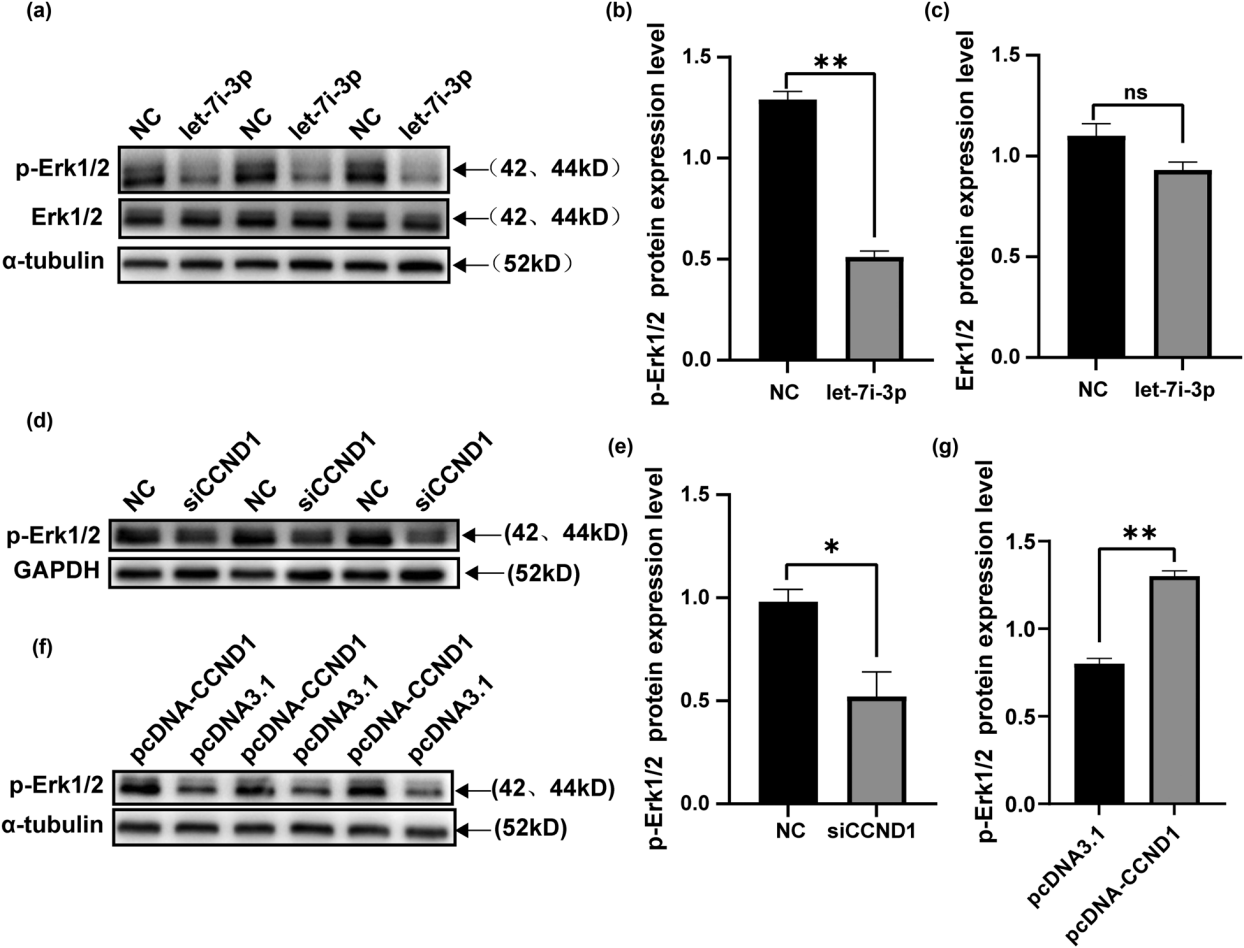
Effects of let-7i-3p and CCND1 on the ERK signaling pathway. (a) Western bolts of Erk1/2 and p-Erk1/2 after the transfection of let-7i-3p mimics or NC in HCT116 cells and statistical histogram was shown at right (b and c). (d) Western bolts of p-Erk1/2 after transfection of siCCND1 or NC in HCT116 cells and statistical histogram was shown at right (e). (f) Western bolts of p-Erk1/2 after transfection of pcDNA-CCND1 or pcDNA3.1 in HCT116 cells and statistical histogram was shown at right (g). *P < 0.05 and **P < 0.01.

To examine the mechanisms of how let-7i-3p and CCND1 inhibited the cell cycle, proliferation, migration, and invasion in CRC; we investigated whether these effects were mediated by activating the ERK signaling pathway. Western blot was used to examine CCND1 expression levels and p-ERK. As shown in Figure 6a and c, overexpressed let-7i-3p caused a significant decrease in p-ERK in HCT116 cells by comparison to the control group. However, there was no significant difference in total-ERK expression. Similarly, the same results can be obtained by downregulating the CCND1 expression in HCT116 cells (Figure 6d and e). We ectopically expressed CCND1 in HCT116 cells that increased significantly the p-ERK compared to the control group (Figure 6f and g). These results suggested that CCND1 played a catalytic role in the ERK signaling pathway.

All these results suggested that CCND1 was a downstream functional regulator of let-7i-3p through ERK signaling pathway.

## 4 Discussion

There are not many studies on let-7i-3p. Luo et al. reported that let-7i-3p inhibited the osteogenic differentiation of hASCs under cyclic strain *in vitro* acting as a negative regulator of the Wnt/β-catenin pathway by targeting LEF1 [19]. Sun et al. observed that low expression of let-7i-3p can enhance the osteoblast differentiation in ankylosing spondylitis (AS) mice by upregulating PDK1 [20]. Falzone et al. proved that let-7i-3p was associated with oral cancer recurrence [21]. Tang et al. reported that microarray data showed let-7i-3p levels significantly reduced in CRC cell lines (SW620, LoVo) compared to normal colon epithelial FHC cells [12]. However, there are few studies on the role of let-7i-3p in the pathogenesis of CRC.

In our study, we confirmed that the expression level of let-7i-3p in CRC cell lines (HCT116, SW480, and LoVo) was significantly lower than that in FHC by qRT-PCR (Figure 1a). Based on bioinformatics software prediction and literature review, we attempted to detect the expression level of the target gene CCND1 in CRC cell lines and FHC. As expected, the result showed that CCND1 levels in CRC cell lines (HCT116, SW480, and LoVo) were significantly higher than that in FHC by qRT-PCR (Figure 1b). Next, we demonstrated a role for let-7i-3p in the cell cycle, proliferation, invasion, and migration of HCT116 cells (Figure 2). Then, we performed a luciferase reporter assay to verify that CCND1 was a direct target of let-7i-3p (Figure 3). To elucidate the mechanism underlying the effects of let-7i-3p on proliferation, migration, and invasion, we tested whether CCND1 was required for the function of let-7i-3p by transfecting CCND1 siRNA into HCT116 cells. Likewise, silencing CCND1 inhibited the cell cycle, proliferation, invasion, and migration of HCT116 cells (Figure 4). Furthermore, ectopic expression of CCND1 offsets the inhibition of let-7i-3p overexpression on cell proliferation, migration, and invasion (Figure 5). These results suggested that CCND1 may act as a target of let-7i-3p and participate in the effect of let-7i-3p on the cell cycle, proliferation, migration, and invasion of CRC cells.

It is well known that the ERK signaling pathway plays an important role in several cellular processes, including cell cycle, proliferation, metastasis, survival, and apoptosis [22]. Leng et al. demonstrated that miR-29b suppressed the EMT and angiogenesis in CRC by disrupting the ETV4-dependent activation of the ERK signaling pathway [23]. Liu et al. revealed that miR-128-3p downregulated the deterioration rate of CRC by simultaneously silencing the activity of PI3K/AKT and MEK/ERK pathway [24]. Based on previous findings, we then investigated the effect of let-7i-3p and CCND1 on the regulation of the ERK pathway. As demonstrated that overexpression of the let-7i-3p resulted in the inhibition of the p-ERK signal in HCT116 cells. Similarly, the same results can be obtained by downregulating the CCND1 expression by siRNA. In contrast, overexpression of CCND1 in the HCT116 cells led to the abnormal activation of the ERK pathway (Figure 6).

In summary, we reported a tumor suppressor for let-7i-3p in CRC progression. We showed that let-7i-3p inhibited cell cycle, proliferation, migration, and invasion in HCT116 cells. We also confirmed that let-7i-3p inhibited the ERK signaling activity through direct suppression of CCND1. Overall, we have identified the role and molecular mechanism of let-7i-3p in HCT116 cells, and let-7i-3p may be a potential target for CRC treatment in the future.

## Supplementary Material

Supplementary Figure
