# Supplementary material for: Let-7i-3p inhibits the cell cycle, proliferation, invasion, and migration of colorectal cancer cells via downregulating CCND1
Source: Open Med (Wars). 2022 Jun 7;17(1):1019–30. doi: 10.1515/med-2022-0499 (PMC9175015; doi:10.1515/med-2022-0499)
Supplement: Supplementary Figure [file med-2022-0499-sm.pdf]

Supplementary material

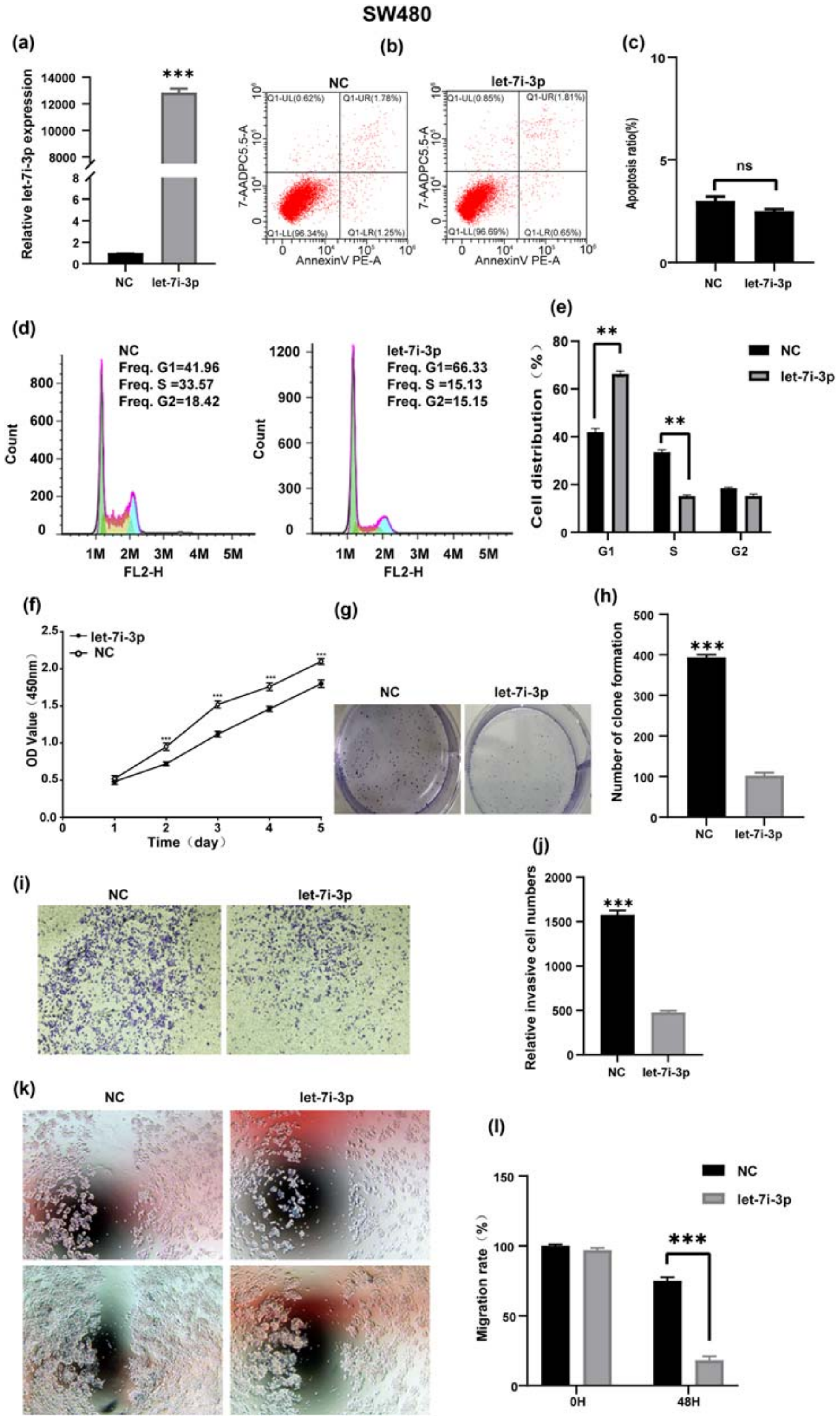

(caption on next page)

Figure S1: Let-7i-3p inhibits the cell cycle, proliferation, migration and invasion, but does not affect the apoptosis in SW480. (a) The expressions of let-7i-3p were measured after transfecting let-7i-3p or NC into SW480 cells. (b and c) Cell viability was determined by Annexin V/7-AAD staining. Representative flow cytometric analysis of apoptosis (B) and statistical histogram was shown at right (c). (d) Relative cell cycle distribution detected by flow cytometry and statistical histogram was shown at right (e). (f) The effects of let-7i-3p mimics or NC on SW480 cells proliferation as determined by CCK-8 assay. (g) Colony formation assay was used to detect cell colony formation ability after transfection of let-7i-3p in SW480 cells and statistical histogram was shown at right (h). (i) The effects of let-7i-3p mimics and NC on SW480 cells invasion determined by transwell assay and statistical histogram was shown at right (j). (k) Images were acquired at 0 and 48 h after wounding. The percentage of the wound healing was calculated as (the width of wound at 0 h - the width of wound at 48 h)/the width of wound at 0 h and statistical histogram was shown at right (L). *$p < 0.05$, **$p<0.01$, ***$p<0.001$.
